# Rapid Immobilization of Simulated Radioactive Soil Waste Using Self-Propagating Synthesized Gd_2_Ti_2_O_7_ Pyrochlore Matrix

**DOI:** 10.3390/ma12071163

**Published:** 2019-04-10

**Authors:** Jiali Xue, Kuibao Zhang, Zongsheng He, Wenwen Zhao, Weiwei Li, Dayan Xie, Baozhu Luo, Kai Xu, Haibin Zhang

**Affiliations:** 1 State Key Laboratory of Environment-friendly Energy Materials, Southwest University of Science and Technology, Mianyang 621010, China; xuejiali0304@163.com (J.X.); hezongsheng@swust.edu.cn (Z.H.); zhaowenwen@swust.du.cn (W.Z.); liweiwei@swust.du.cn (W.L.); xiedayan@swust.edu.cn (D.X.); luobaozhu@swust.edu.cn (B.L.); 2 Sichuan Civil-Military Integration Institute, Mianyang 621010, China; 3 State Key Laboratory of Silicate Materials for Architectures, Wuhan University of Technology, Wuhan 430070, China; kaixu@whut.edu.cn; 4 Institute of Nuclear Physics and Chemistry, China Academy of Engineering Physics, Mianyang 621900, China

**Keywords:** Radioactive soil waste, Gd_2_Ti_2_O_7_ pyrochlore, SHS, CeO_2_, Immobilization

## Abstract

A rapid and effective method is necessary in the disposal of severely radioactive contaminated soil waste. Simulated Ce-bearing radioactive soil waste was immobilized by self-propagating high-temperature synthesis (SHS) within 5 min in this study. The main work includes the rapid synthesis of soil waste forms, the analysis of phase composition, microstructure and chemical durability. These results show that the simulated nuclide Ce was successfully immobilized into the pyrochlore-rich waste matrice, whose main phases are SiO_2_, pyrochlore (Gd_2_Ti_2_O_7_) and Cu. The normalized leaching rates of Si and Na on the 42nd day are 1.86 × 10^−3^ and 1.63 × 10^−2^ g·m^−2^·d^−1^, respectively. And the normalized leaching rate of Ce also remains at low level (10^−5^–10^−6^ g·m^−2^·d^−1^) within 42 days.

## 1. Introduction

In recent years, nuclear power has been developed rapidly in the world due to its advantages of high efficiency, economy and low carbon emissions. However, the harm caused by the byproduction of nuclear energy, mainly nuclear wastes, can hardly be ignored, especially high-level radioactive waste (HLW) [1]. The radioactive nuclides in HLW, such as ^137^Cs, ^90^Sr, ^239^Pu, ^235^U, etc., possess the characteristics of long half-life, high toxicity, and high heat generation [2,3]. When these radionuclides enter the soil, the situation becomes more complicated because the presence of soil will increase the cost of disposal [4,5]. Furthermore, soil contaminated by highly radioactive nuclides may pose a long-term threat to organisms due to ecological cycling [6,7].

For radioactive contaminated soil, sand is an inseparable main substance and must be cured together with radionuclides. At present, there are mainly physical landfill and bioremediation methods for the treatment of radioactive contaminated soil. The physical landfill is a time-consuming project, which will destroy the ecosystem of disposal area. At the same time, it may cause further pollution due to leakage during transportation [8]. The bioremediation method requires a long period of restoration, and the growth of plants is limited by climate and geology [9]. In addition to the above methods, vitrification is an effective technology for the immobilization of long half-life wastes, especially for soil wastes contaminated by high radioactive nuclides [10,11]. Particularly, borosilicate glass is the most widely studied and utilized vitrified waste form in the world because of its good radiation resistance, corrosion resistance, and chemical stability [12]. Regrettably, the glass matrice may decompose in geological repositories due to high temperature and high pressure [13,14]. Furthermore, the relatively low thermal stability of vitrified waste forms is also a potential limitation [15]. Compared with glass matrice, ceramic solidified bodies have the advantages of low expansion rate, excellent mechanical properties, and good chemical stability. Subsequently, Synroc has been proposed as a potential alternative host matrix for HLW immobilization based on the isomorphism substitution theory [16,17,18,19,20].

Self-propagating high-temperature synthesis (SHS) is a technology that uses the energy released by exothermic redox reactions to synthesize the final products [21,22,23]. SHS technology possesses certain technical and cost advantages in the treatment of contaminated soil. According to the characteristics of radionuclides, the composition and proportion of a SHS reaction system can be well designed. In addition, quick pressing (QP) is also introduced to obtain compact samples [24]. Zirconolite-rich matrice and titanate-pyrochlore with excellent chemical durability can also be prepared by SHS/QP [25,26,27,28,29,30]. SHS/QP technology can synthesize high density ceramic matrix in several minutes, which was considered as a potential method to deal with environmental issues. In this study, Gd_2_Ti_2_O_7_ pyrochlore waste matrix was synthesized by SHS for the disposal of simulated radioactive soil waste. Ten wt.% CeO_2_ was regarded as a simulate of tetravalent actinide [31]. Silica sand was utilized as the heat insulation material and pressure transfer medium during the SHS/QP process. A series of characterizations were carried out to understand the solidifying mechanism of obtained waste forms. In addition, the aqueous durability was evaluated using the standard Product Consistency Test (PCT) method [32].

## 2. Materials and Methods

The composition of original soil is listed in Table 1. The soil (200 meshes) and simulated radionuclide Ce^4+^ (CeO_2_, Aladdin Industrial Inc., purity ≥ 99.99%)) were mixed with the weight ratio of 9:1. The SHS reaction was prepared according to the following chemical equation [28]:

4CuO + Gd_2_O_3_ + 2Ti = Gd_2_Ti_2_O_7_ + 4Cu (1)

The raw materials of CuO, Gd_2_O_3_, and Ti (purity ≥ 99.9 wt.%) were purchased from Aladdin Industrial Inc. (Shanghai, China). Different contents of simulated radioactive soil wastes (0 wt.%, 5 wt.%, 10 wt.%, 15 wt.%, 20 wt.%, 25 wt.%) were mixed with the raw materials of SHS reaction (labelled as Cu-0, Cu-5, Cu-10, Cu-15, Cu-20, and Cu-25, respectively). Pretreatment of powder samples is similar as the previous study [28].

**Figure 1:**
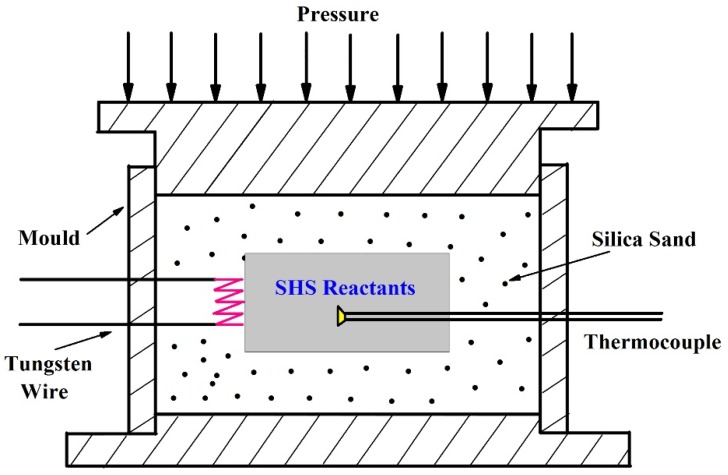
Diagrammatic sketch of the self-propagating high-temperature synthesis/quick pressing (SHS/QP) process.

The SHS/QP process is shown in Figure 1. The self-propagating combustion reactions were ignited by tungsten wire, which was located at one side with tight contact of the green body and heated by a direct current of about 50 A. The SHS reactants were ignited at high temperature, and the combustion wave automatically propagated to the unreacted region until the reaction’s completion. Before densification, the W/Re 5/26 thermocouple was placed in the center of the samples to measure the reaction temperature of Cu-0 to Cu-25 specimens. The unpressurized samples were crushed into fine powders for X-ray diffraction analysis (XRD; X’Pert PRO, PANalytical B.V., Almelo, The Netherlands).

For densification, the ignited sample was compressed by 50 MPa with 60 s dwelling time after proper combustion delay time. The SHS-ed compact sample was cut and polished to characterize the microstructure and elemental distribution using field-emission scanning electron microscopy (FESEM; Zeiss Ultra-55, Oberkochen, Germany) and energy-dispersive X-ray spectroscopy (EDX, ULTRA 55, ZEISS, Oberkochen, Germany). The chemical durability of waste form was tested by the Product Consistency Test (PCT) standard. The concentrations of Na and Si in leachate were determined by inductively coupled plasma (ICP) analysis (iCPA 6500, ThermoFisher, Waltham, MA, USA), while that of Ce was obtained by inductively coupled plasma-mass spectrometry (ICP-MS) analysis using an Agilent 7700× spectrometer (Agilent, Santa Clara, CA, USA). The normalized release rates were calculated as the following formula:

(2) $${NR}_{i}\;=\;\frac{C_{i}\cdot V}{f_{i}\cdot S_{A}\cdot t}$$

where *C_i_* is the concentration of element *i* in the solution, *V* is the volume of the leachate (m^3^), *S_A_* is the surface area of powder specimen (m^2^), *f_i_* is the mass fraction of element *i* in the sample (wt.%) and *t* is the leaching duration (d). The *S_A_/V* ratio is about 2000 m^−1^, which is derived from the standard test method for The Product Consistency Test (ASTM c 1285-02) [32]. In this standard, the waste particles are assumed to be spherical and the average particle diameter is 1.12 × 10^−4^ m for −100 (0.149 mm) to +200 (0.074 mm) meshes particles. Therefore, the average particle area and volume are calculated as 3.90 × 10^−8^ m^2^ and 7.25 × 10^−13^ m^3^, respectively. The average particle mass is calculated to be 1.96 × 10^−6^ g. Thus, there are 1 g/1.96 × 10^−6^ g = 5.11 × 10^5^ particles in 1 g powder waste form with −100 to +200 meshes particles. Thus, the total surface area of 1 g powder with −100 to +200 meshes particles is calculated to be 1.99 × 10^−2^ m^2^. As long as the density and particle size of waste form remain comparable during the leaching tests, this parameter will remain at a constant value and doesn’t need to be calculated every time.

## 3. Results and Discussion

### 3.1. Temperature and Powder XRD Analysis

**Figure 2:**
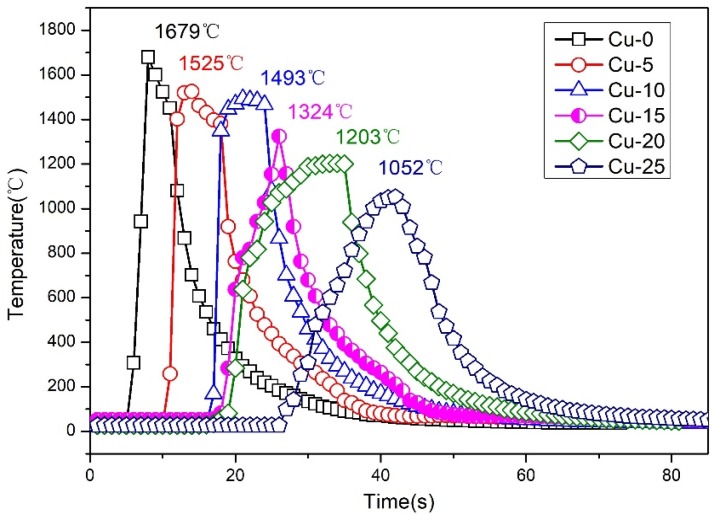
Real temperature curves of all samples during SHS reaction.

The combustion process of the designed SHS reaction takes about 10 s after tungsten wire ignition. The center temperature of all samples in SHS reactions are measured and depicted in Figure 2. With the increment of soil wastes, the center temperatures of Cu-0, Cu-5, Cu-10, Cu-15, Cu-20, and Cu-25 samples decrease from 1679 to 1052 °C in Figure 2. The center temperature of the Cu-0 sample is the highest at 1679 °C, while the Cu-25 sample with the maximum soil content exhibits the lowest temperature at 1052 °C. Apparently, the increase of soil wastes led to the decrease of SHS reaction temperature.

**Figure 3:**
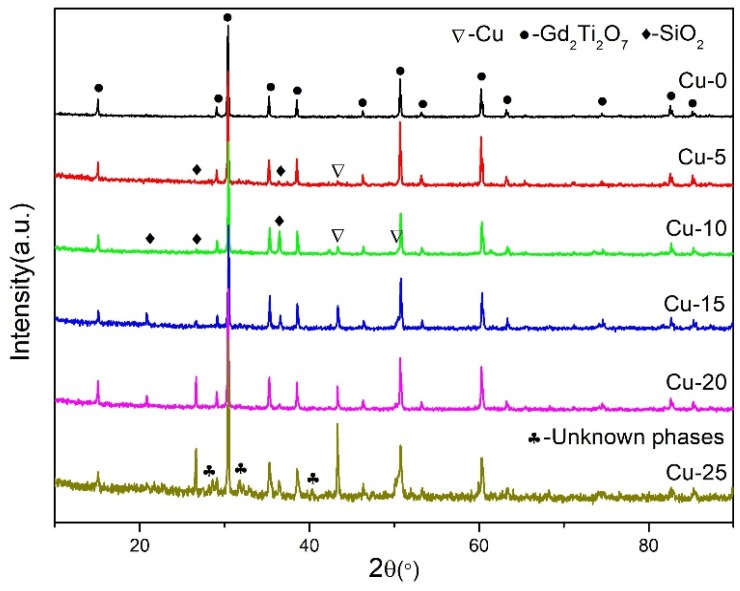
X-ray diffraction (XRD) patterns of all SHS-ed samples.

The XRD patterns in Figure 3 show that the specimens with soil waste (Cu-5 to Cu-25 samples) are composed of Cu, SiO_2_, and Gd_2_Ti_2_O_7_ (PDF No. 23-0259), while the sample without soil waste only contains Gd_2_Ti_2_O_7_ pyrochlore. From Cu-5 to Cu-25 specimens, the main phase of all samples is Gd_2_Ti_2_O_7_ pyrochlore, demonstrating that the increase of soil wastes does not change the phase composition. In Figure 3, the content of Cu in these SHS-ed samples increases with the increment of soil content, but Cu is hardly found in the Cu-0 sample. Because the temperature of Cu-0 reaction is the highest, the Cu melts and condenses into bulky grains during the high temperature reaction. With the decrease of reaction temperature, the size of copper particles decreases. Meanwhile, all SHS-ed samples were ground into powder for XRD testing, where the granulated Cu was sifted out directly. By contrast, the content change of SiO_2_ has no regular pattern, which may be affected by the heat insulator silica sand. However, unknown phases appear in the Cu-25 sample, which may be related with the large amount of simulated radioactive soil. Therefore, the Cu-20 specimen was selected for further analysis.

### 3.2. Raman Analysis and Microstructure Characterization

Raman spectroscopy was carried out to further analyze the crystal structure and internal bonds of pyrochlore. Raman spectroscopy is an important technique, especially in systems where oxygen displacement induces structure transformation, such as distinguishing fluorite from pyrochlore in pyrochlore ceramics [33]. Different from the A_2_B_2_O_7_ fluorite structure with only one F_2g_ vibration mode, the pyrochlore structure contains six Raman modes (A_1g_, E_g_, and 4F_2g_). Typical wavenumbers of pyrochlore phase at room temperature are 520 cm^−1^ (A_1__g_), 330 cm^−1^ (E_g_), and 200, 310, 450, 580 cm^−1^ (4F_2__g_) [33,34]. For Ti-pyrochlore, the most prominent characteristic of Raman spectra are the intensive band at 320 cm^−1^ and the A_1_*_g_* band at 520 cm^−1^. The band around 320 cm^−1^ includes E_g_ + F_2g_ modes with very close frequency, which is mostly attributed to O–A–O bond vibration. The A_1_*_g_* band at 520 cm^−1^ is believed to be related to A–O stretching [35,36].

**Figure 4:**
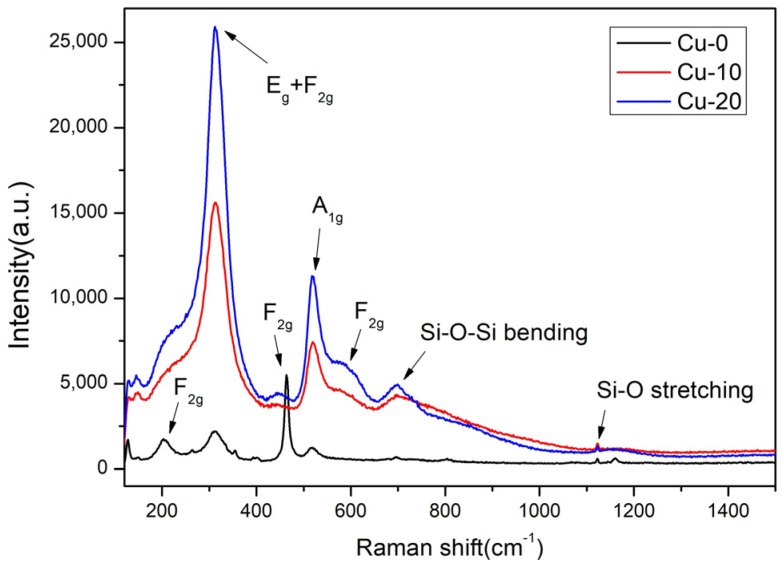
Raman spectra of the Cu-0, Cu-10, and Cu-20 samples.

The Raman spectra of Cu-0, Cu-10, and Cu-20 samples are shown in Figure 4. The six Raman active vibration modes (A_1g_, E_g_ and 4F_2g_) are explicitly assigned. In addition, the Si–O stretching vibration at 1100 cm^−1^ and the Si–O–Si symmetric bending vibration near 700 cm^−1^ are also included. The Raman spectra peaks of three specimens are similar except for some changes in strength, which means the pyrochlore structure of Gd_2_Ti_2_O_7_ remains unchanged. In particular, the characteristic F_2g_ (200 cm^−1^ and 455 cm^−1^) bands are well defined in the Cu-0 specimen. On the contrary, the vibration intensity of E_g_ + F_2g_ modes (320 cm^−1^) and A_1g_ mode (520 cm^−1^) increase significantly in the Cu-10 and Cu-20 samples. It is evident that this drastic change is due to the addition of simulated radioactive soil. On the basis of previous literatures [33,34,35,36], we preliminarily speculate that some ions in the simulated radioactive soil (possibly containing Ce) occupy the A and B sites of pyrochlore structure, resulting in steep changes of oxygen ions’ environment and peak intensity of Raman spectra.

**Figure 5:**
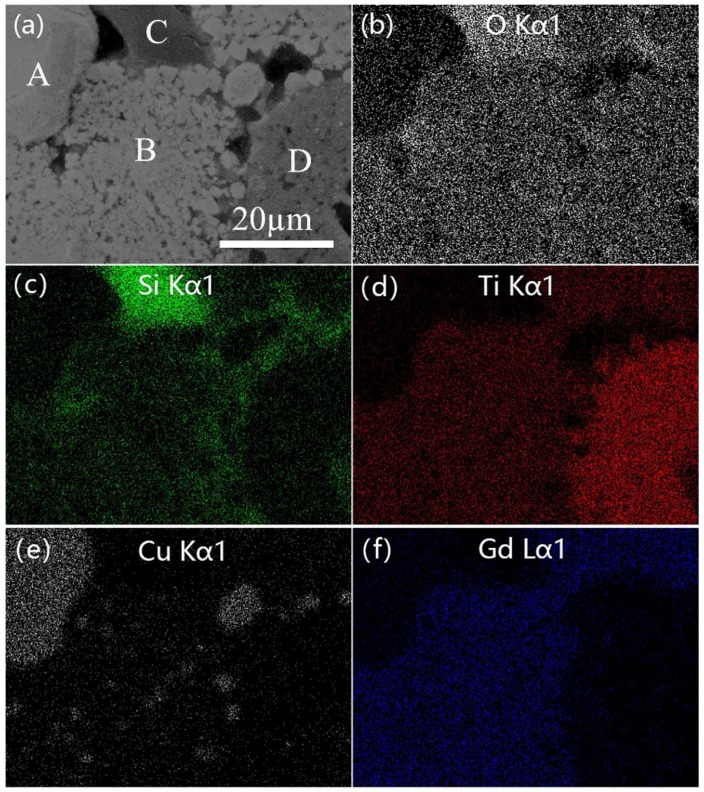
(**a**) SEM image of Cu-20 specimen, and element mapping images of (**b**) O, (**c**) Si, (**d**) Ti, (**e**) Cu, (**f**) Gd.

As shown in Figure 5, the microstructure and elemental distribution of the compact Cu-20 specimen are exhibited in the SEM and elemental mapping images. It can be found that the pores mainly exist in the ceramic matrix rather than the copper phase. It may be argued that the melting point of copper (1083.4 °C) is lower than the combustion temperature of the Cu-20 sample. Therefore, gas can easily be discharged from the copper into the ceramic matrix. The Cu-20 sample consists of four phases, labeled as A, B, C, D in Figure 5a. According to Figure 5b–f and XRD analysis, we speculate that the A region is copper, the B region should be Gd_2_Ti_2_O_7_, the C region is SiO_2_, and the D region represents TiO_2_. The impurity TiO_2_ phase is produced from the raw materials of the reaction system and the original soil. However, no TiO_2_ exists in the previous XRD result of Cu-20. It is possible that the diffraction peaks of TiO_2_ are not obvious because of its low content.

**Figure 6:**
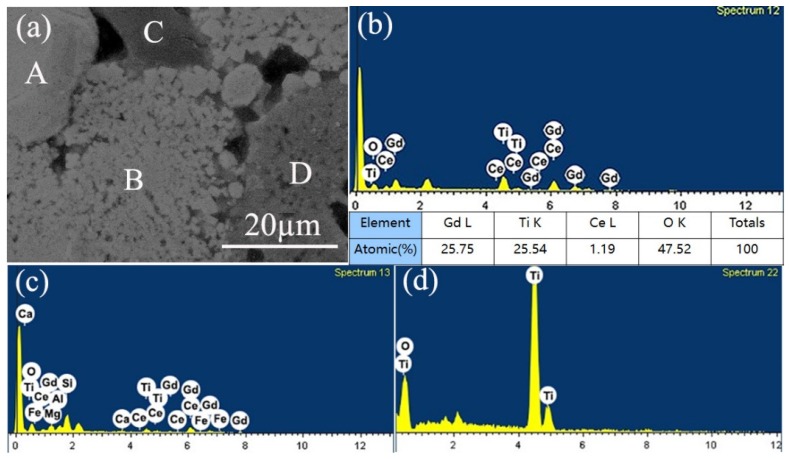
Energy dispersive X-ray spectroscopy (EDX) elemental spotting analysis: (**a**) Representative SEM image of the Cu-20 sample, (**b**) EDX spectrum and elemental composition of the labeled “B” area in (**a**), (**c**) EDX spectrum of region C in (**a**), (**d**) EDX spectrum of region D in (**a**).

The EDX elemental spotting analysis of the Cu-20 sample is presented in Figure 6. The EDX spotting image of “B” phase in Figure 6a is presented in Figure 6b. Combined with the XRD and EDX mapping results, the existence of Gd, Ti, Ce, and O in the EDX spotting spectra indicates that the “B” phase is Ce doped Gd_2_Ti_2_O_7_ pyrochlore phase. The average elemental quantities are acquired by taking at least five points of “A” area as listed in the inserted table of Figure 6b, which results in the chemical formulation of Gd_1.96_Ti_1.94_Ce_0.09_O_7_. Meanwhile, a small amount of Ce is also found in the soil phase according to Figure 6c, indicating that the simulated nuclide Ce of radioactive soil waste can exist in both the pyrochlore phase and soil phase. At the same time, most of the elements in original soil are retained in the soil phase. Figure 6d shows that only Ti and O are present in the D region, which confirms that the D phase is TiO_2_.

### 3.3. Chemical Durability Measurement by PCT Leaching Test

**Figure 7:**
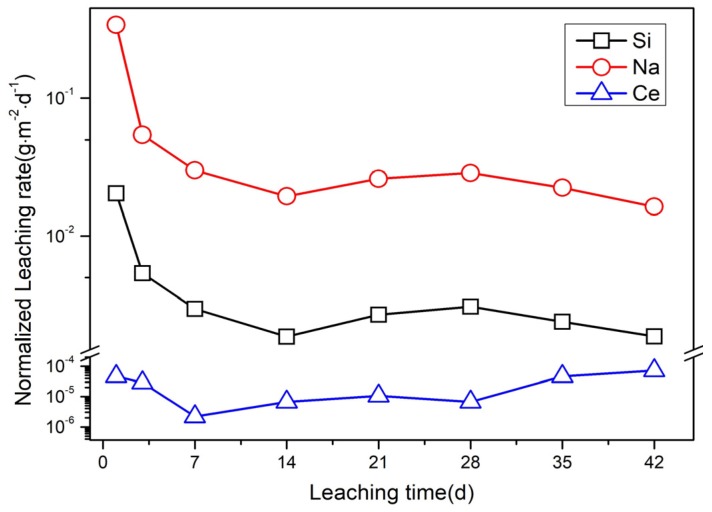
Normalized leaching rates of Si, Na, and Ce from day 1–42.

The leaching performance of nuclear waste forms is a significant indicator for estimating the chemical stability [37]. The 1–42 days normalized elemental leaching rates of Si, Na, and Ce of the Cu-20 sample are depicted in Figure 7. With the extension of soaking time, the normalized leaching rates of Na and Si show a downward trend from 1 to 14 days. Then, *NR*_Na_ and *NR*_Si_ exhibit slight ascension within 14–28 days, and finally both of them reach the lowest values in 42 days. The day 1 and day 42 *NR*_Si_ values are 2.04 × 10^−2^ and 1.86 × 10^−3^ g·m^−2^·d^−1^, which represent excellent stability. The lowest value of *NR*_Na_ is 1.63 × 10^−2^ g·m^−2^·d^−1^ after 42 days of leaching. This rule is not applicable for Ce, whose leaching rate has no definite regulation. The *NR*_Ce_ varies from 7.20 × 10^−5^ g·m^−2^·d^−1^ of 42 days to 2.20 × 10^−6^ g·m^−2^·d^−1^ of 7 days, which is about five orders of magnitude lower than the *NR*_Na_ values. The *NR*_ce_ values vary irregularly but remain at a low level (10^−5^–10^−6^ g·m^−2^·d^−1^). Compared with our previous research results [38], the leaching rate of Si is slightly lower in this study. The *NR*_Na_ of the Cu-20 sample is similar to that of borosilicate glass-ceramics [39]. Nevertheless, the leaching performance of simulated nuclide in Cu-20 sample is significantly lower than that of typical vitreous products [40].

## 4. Conclusions

In summary, a series of Gd_2_Ti_2_O_7_-based waste forms containing 5–25 wt.% simulated radioactive contaminated soil have been successfully synthesized by SHS in 5 min. The obtained products are multiphase composite materials composed of SiO_2_, Gd_2_Ti_2_O_7_, and Cu. Furthermore, the simulated nuclide Ce exists in pyrochlore and soil phases simultaneously, which indicates that Ce migrates partly from soil to pyrochlore phase during the SHS reaction. The solidified body of Cu-20 sample exhibits high stability. The 42 days *NR*_Si_ and *NR*_Na_ are as low as 1.86 × 10^−3^ and 1.63 × 10^−2^ g·m^−2^·d^−1^, respectively. And the 1–42 days *NR*_Ce_ values also remain at a low level (10^−5^–10^−6^ g·m^−2^·d^−1^). Based on the analysis of phase composition, microstructure, and chemical durability, the application potential of SHS technology in the rapid disposal of radioactive soil wastes is revealed.

## Figures and Tables

**Table 1:** Soil composition in this study.

Composition	SiO_2_	Al_2_O_3_	Fe_2_O_3_	CaO	K_2_O	MgO	Na_2_O	TiO_2_
Content (wt.%)	66.32	16.57	5.87	4.67	2.86	1.66	0.81	0.74
